# Exosomal AFAP1-AS1 binds to microRNA-15a-5p to promote the proliferation, migration, and invasion of ectopic endometrial stromal cells in endometriosis

**DOI:** 10.1186/s12958-022-00942-1

**Published:** 2022-05-05

**Authors:** Xi Wang, Mengmeng Zhang, Liaofei Jiang, Xiaoling Fang, Tingting Zhang

**Affiliations:** 1 Department of Obstetrics and Gynecology, The Second Xiangya Hospital, Central South University, NO.139 Renmin Road, Changsha, Hunan 410000 PR China; 2 Department of Obstetrics and Gynecology, Women’s Hospital, School of Medicine, Zhejiang University, Hangzhou Zhejiang, 310006 PR China

**Keywords:** Endometriosis, Exosome, Long non-coding RNA actin filament associated protein 1-antisense RNA 1, microRNA-15a-5p, B-cell lymphoma 9, Proliferation, Endometrial stromal cells

## Abstract

**Background:**

Endometriosis (EMS) remains a major challenge to reproductive health due to multifactorial etiology, disease heterogeneity, and the lack of appropriate diagnostic markers and treatment. Eexosome (Exo) has become a major factor in progression of a variety of diseases. However, the mechanisms directing their role in the pathophysiology of EMS are ill-defined. Here, we aimed to investigate the clinical implications of actin filament associated protein 1-Antisense RNA 1 (AFAP1-AS1) in EMS.

**Methods:**

Bioinformatics analysis was used to predict the expression and interaction of AFAP1-AS1, miR-15a-5p and BCL9 in EMS, and dual luciferase reporter assay was used to verify the targeted relationship of AFAP1-AS1, miR-15a-5p, and BCL9. The Exo from endometrial stromal cells (ESCs) was isolated and characterized by transmission electron microscopy (TEM) and Nanoparticle tracking analysis (NTA). Exosome uptake studies were performed. For in vitro assay, ectopic ESCs (EcESCs) proliferation, migration, and invasion were assessed by CCK-8 and Transwell assays. In vivo assay was performed by establishment of EMS mice to validate the result derived from in vitro assay.

**Results:**

The Exo was successfully isolated from ESCs and we observed high expression of AFAP1-AS1 and BCL9 but low expression of miR-15a-5p in EMS. Moreover, Exo derived from EcESCs could deliver AFAP1-AS1 to EcESCs and thus promoting proliferation, migration, and invasion of ESCs. AFAP1-AS1 bound to BCL9, which was targeted by miR-15a-5p in EMS. In vivo experiments in nude mice revealed that inhibition of Exosomal AFAP1-AS1 suppressed migration and invasion of EcESCs through miR-15a-5p/BCL9.

**Conclusions:**

Collectively, these findings suggested that ESCs-derived Exo carrying AFAP1-AS1 contributed to EMS pathogenesis. This study might help us realize the etiology of EMS and improve the treatment of the related complications.

**Supplementary Information:**

The online version contains supplementary material available at 10.1186/s12958-022-00942-1.

## Background

Endometriosis (EMS) usually occurs during the growth of endometrial tissue outside the uterine cavity and ectopic implantation [1]. EMS is frequently seen among women, seriously making negative effects on their quality of life, while the etiology and pathogenesis of EMS are still largely unknown [2]. The identification of endometrial stromal cells (ESCs) in human endometrium has inspired an area of research on EMS [3]. The proliferation, migration, and invasion of ESCs might be a novel potential therapeutic approach to the treatment of EMS, which may shed new light on the understanding of the pathogenesis of EMS [4]. Also, exosome (Exo) is a small extracellular vesicle with diameters of 40-100 nm, which is often secreted by a variety of living cells and contains releasable functionally active proteins, mRNA, as well as microRNAs (miRNAs), being involved in diseases related to infertility [5]. The transfer of long noncoding RNAs (lncRNAs) via Exo to recipient cells represents is considered as a mechanism for EMS progression [6]. Of noted, Exo derived from ESCs plays an autocrine or paracrine role in EMS [7], which warrants in-depth investigation.

LncRNA actin filament associated protein 1-Antisense RNA 1 (AFAP1-AS1) is an oncogenic lncRNA that can be able to promote proliferation, invasion and migration of tumor cells [8]. AFAP1-AS1 was correlated with endometrial carcinoma patients’ clinical characteristics and prognosis, which could affect the cell activities and contributed to endothelial cell angiogenesis in endometrial carcinoma [9]. Furthermore, AFAP1-AS1 has been studied in EMS lesions, while its detailed mechanism of progression remains unclear [10]. Additionally, miRNAs are modulators of gene expression, and their stability makes them a candidate biomarker as well as a potential non-invasive target to diagnose EMS [11]. miR-15a-5p is lowly expressed in EMS and that overexpression of miR-15a-5p inhibits the proliferation and migration of Ectopic (EcESCs) [12]. miR-15a-5p can mediate endometrial cancer cell proliferation by targeting Wnt3a [13]. In addition, our bioinformatics analysis result showed that miR-15a-5p could mediate B-cell lymphoma 9 (BCL9), while the relevant regulatory mechanism was still unclear. BCL9 is an oncogene in EMS [14], yet the specific mechanism remains unclear. Our hypothesis is that Exo released from ESCs participates in the regulation of EMS progression by delivering specific lncRNA. In this study, we have established both in vitro and in vivo assays to prove the function of exosome delivery for proliferation, migration, and invasion of ESCs.

## Materials and methods

### Ethical approval

All tissue samples were obtained with full and informed patient consent, and all experiments were followed the ethical principles outlined by the 1964 Helsinki Declaration and its later amendments or comparable ethical standards. This study was approved by institutional ethics review board of the Second Xiangya Hospital, Central South University.

### Clinical sample collection

In this study, snap-frozen cyst walls of ovarian endometriomas and matched eutopic endometrium of the uterus, from the same patient who underwent laparoscopy, were simultaneously collected (20 to 40 years of age, proliferative phase, *n* = 30, revised classification of American Fertility Society, r-AFS stage III/IV). Paired specimens, selected randomly, and twenty normal endometrium from women free of endometriosis (control group) (20 to 40 years of age, proliferative phase, *n* = 20) were as controls. All the patients had regular menstrual cycles and did not receive hormonotherapy or immunosuppressor for at 6 months prior to the specimen collection. Furthermore, patients in the control group had no evidence of tumor in the endometrium and did not have histological diagnosis of adenomyosis. The specimen diagnosis was determined by histopathology and the menstrual cycle was confirmed by both last menstrual period and histological ascertain. Some of the tissue specimens were fixed in formalin and some were stored at − 80 °C.

### Second-generation sequencing

Samples of eutopic and ectopic endometrial tissues were taken from three EMS patients. Total RNA was isolated using Trizol reagent (15,596,018, Invitrogen, Calsbad, CA, USA) according to the manufacturer’s protocol. RNA sample concentrations were determined by OD260/280 using a Nanodrop ND-1000 spectrophotometer (Thermo Fisher Scientific, Waltham, MA, USA). RNA integrity was checked by agarose gel electrophoresis at a 28S:18S ratio of ≥1.5. miRNA sequencing libraries were An Illumina TruSeq® Small RNA Library Prep Kit. A total of 5 μg of RNA was used in per sample, which was quantified and checked using the KAPA library quantification kit (KAPA Biosystems, Wilmington, MA, USA). Paired-end sequencing was performed on an Illumina NextSeqCN500 sequencer. High-throughput sequencing of EMS eutopic and ectopic endometrial tissue samples was performed to screen for differentially expressed miRNAs in EMS patients.

### Bioinformatics analysis

GSE7846 microarray data were obtained from the Gene Expression Omnibus (GEO) database (https://www.ncbi.nlm.nih.gov/gds), a dataset consisting of endometrial cells from five non-EMS patients and five EMS patients. Differentially expressed genes (DEGs) between the EMS and non-EMS samples were analyzed using the R language “limma” package (http://www.bioconductor.org/packages/release/bioc/html/limma.html), with *p* value < 0.05 as the threshold value.

### Isolation and cultivation of ESCs

EcESCs, and normal human ESCs (hESCs) were isolated from the above collected tissues. The tissue samples were added with 3 times the volume of 0.25% type IV collagenase-trypsin ethylenediaminetetraacetic acid (EDTA; Sigma-Aldrich Corporation, St. Louis, MO, USA) solution and pre-warmed to 37 °C. After digestion, the tissue solutions were filtered through 150 μm and 74 μm stainless steel cell filters (GongLu; Hangzhou, China) to obtain cells, respectively. These cells were finally cultured in Dulbecco’s Modified Eagle Medium (DMEM)/F-12 (A4192001, Gibco, Waltham, CA, USA) containing 10% fetal bovine serum (FBS, 16140071, Gibco) and 1% antibiotic-antifungal solution (100 ×, 15,240,112, Gibco). After purification, the morphology was observed by immunofluorescence staining and the expression of vimentin in the cells was identified by immunofluorescence and immunohistochemistry (IHC).

For Transwell co-culture system, EcESCs, dimethyl sulfoxide (DMSO) and the sphingomyelin inhibitor GW4869 (10 μM, Sigma-Aldrich) were added to the apical chamber, and EcESCs were added to the basolateral chamber for 24 h. Cells were differently treated by DMSO or GW4869.

### Immunofluorescence

Primary and passaged ESCs were seeded onto sterile slides, and the adherent cells were removed after incubation, washed in phosphate buffer saline (PBS), fixed in 4% paraformaldehyde for 20 min, and blocked for 2 h in blocking solution. Cells slides were incubated for 3–5 h at room temperature with Vimentin primary antibody (1/200, 5741, Cell Signaling Technology, Hercules, CA, USA), and then with fluorescence secondary antibody immunoglobulin G (IgG) H&L (ab6785, 1:100, Abcam, Cambridge, UK) in the dark room. Cell nuclei were stained with 4′,6-Diamidine-2-phenylindole dihydrochloride (DAPI, C1025, Beyotime, Nantong, China) for 15 min, and cell morphology and Vimentin expression were observed under fluorescence microscope.

### Cell transfection

Cells were transfected with the plasmids of short hairpin RNA against AFAP1-AS1 (sh-AFAP1-AS1), BCL9 overexpression vector (oe-BCL9), or their corresponding negative control (sh-NC or oe-NC). Expression vectors were transfected into 293 T cells separately using the Lipofectamine 2000 (Invitrogen), and the supernatant was collected to obtain the viral solution. For transfection, EcESCs (1 × 10^6^) were seeded in 6-well plates with 2 mL of medium per well, and infection was performed when cell confluence reached 50%. Separately, 800 μL of fresh viral solution was mixed with 800 μL of complete medium, while Polybrene (6 μg/mL, TR-1003-G, Sigma-Aldrich) was added. At 12 h after transfection, fresh complete medium was added and cells were further cultured at 37 °C in 5% CO_2_. Cells were placed in medium containing puromycin (1 μg/mL, A1113803, Thermo Fisher Scientific) 48 h after infection to screen stably transfected cell lines. Cells were collected when they were mortal in the puromycin-containing medium and overexpression or silencing efficiency was confirmed by reverse transcription quantitative polymerase chain reaction (RT-qPCR) or Western blot analysis.

Cells were transfected with plasmids of miR-15a-5p mimic, miR-15a-5p inhibitor or the matched NC. Cells were seeded into six-well plates and transfected with the above plasmids (50 nM; GenePharma, Shanghai, China) according to the Lipofectamine 2000 reagent instructions. Cells were collected 48 h after transfection.

### Isolation and identification of Exo

Exo was obtained from the supernatant of EcESCs medium using the Exo Extraction Kit (ExoQuick; SBI, CA, USA) according to the instructions. When EcESCs confluence reached 90% in the culture dish, the medium was replaced with Exo-free 1640 medium containing FBS. After 24 h of incubation, the cell culture medium supernatant was collected from 20 mL of medium (1 × 10^7^ cells) and centrifuged at 3000×g for 15 min. Then 1000 μL of ExoQuick Exosome precipitation solution was added to 1000 μL of supernatant and the mixture was frozen at 4 °C for 30 min. After centrifugation at 1500 g for 30 min, the Exo precipitate was resuspended with 100 μL of sterile 1× PBS and its supernatant was used as a control without Exo. Exo morphology was subsequently observed by transmission electron microscopy (TEM) and the Exo protein markers CD9, CD81, CD63, and Tsg101 were identified by Western blot analysis.

### Transmission electron microscopy (TEM)

After the separation of Exo, images of Exo were taken using TEM. Exo was resuspended and fixed in 30 μL of 2% paraformaldehyde and then adsorbed onto a discharged copper grid. Exo was stained after addition of 4% uranyl acetate. Exo images were acquired by a Hitachi H7650 TEM (Japan).

### Nanoparticle tracking analysis (NTA)

A total of 20 μg Exo was dissolved in 1 mL PBS and vortexed for 1 min to maintain a uniform distribution of Exo, followed by measurement of Exo size distribution using a NTA (Malvern Instruments Ltd., Malvern, UK).

### Internalization of Exo

Exo was labeled with the membrane labeling dye PKH67 green fluorescence (HR8569, BjBalb, Beijing, China). Exo secreted by control cells was labeled using PKH67 dye, and the labeled Exo was co-cultured with EcESCs for 24 h, after which the cells were fixed with 4% paraformaldehyde and the nuclei were stained with 10 μg/mL of DAPI staining solution (C1025, Beyotime) for 10 min. The uptake of labelled Exo by recipient cells was observed using a Nikon Eclipse fluorescence microscope (Nikon, Tokyo, Japan).

### RT-qPCR

Total RNA was extracted from cells, Exo or tissues using TRIzol® reagents (15,596,018, Invitrogen), while lncRNA and mRNA levels were detected by reverse transcription using the PrimeScript™ RT-qPCR kit (RR047A, Takara, Japan). Reverse transcription was performed to detect miRNA levels using the PrimeScript™ miRNA RT-qPCR kit (B532451, Sangon Biotech, Shanghai, China). The SYBR® Premix Ex Taq™ II kit (RR820A, Takara) was used for sample configuration and samples were subjected to real-time PCR. Glyceraldehyde-3-phosphate dehydrogenase (GAPDH) was used as an internal reference for lncRNA and mRNA while U6 was used as an internal reference for miRNA. The primers used for amplification were provided by General Biotechnology (Shanghai, China). Primer sequences are listed in Supplementary Table 1. The relative transcript levels of the target genes were calculated using the relative quantification method (2-^ΔΔCT^ method), in which mRNA relative transcript levels of the target genes = 2-ΔΔCt. Three replicate wells were set up for each sample and each set of experiments was repeated 3 times.

### Western blot analysis

Cells were digested with trypsin and collected. Cells were lysed with enhanced radio immunoprecipitation analysis (RIPA) lysis buffer containing protease inhibitors (AR0102, Boster, Wuhan, China) and protein concentrations were determined using the bicinchoninic acid (BCA) protein quantification kit (AR0146, Boster). The proteins were separated by sodium dodecyl sulfate polyacrylamide gel electrophores (SDS-PAGE), which were electrotransferred to polyvinylidene fluoride (PVDF) membranes. The membrane was sealed with 5% BSA for 1 h at room temperature and then incubated with diluted primary antibodies of CD9 (1/1000, ab195422, Abcam), CD81 (1/1000, ab109201, Abcam), CD63 (1/1000, ab134045, Abcam), Tsg101 (1/1000, ab83, Abcam), Calnexin (1/1000, ab22595, Abcam), BCL9 (1/1000, 22,947–1-AP, Proteintech, VA, USA), and α-tubulin (1/2000, ab52866, Abcam) overnight at 4 °C. The membranes were added with horseradish peroxide (HRP)-labelled goat anti-mouse secondary antibody (1:2000, ab6808, Abcam) for 1 h at room temperature. An appropriate amount of enhanced chemiluminescence (ECL) working solution (AR1174, Boster) was added. The transfer film was incubated at room temperature for 1 min, and X-ray film was placed in the dark box for 5–10 min before development and fixation. The bands in the Western blot images were quantified in grey scale using Image J analysis software, and α-tubulin was used as an internal reference.

### Cell counting kit-8 (CCK-8)

Cell proliferation capacity was assessed using the CCK-8 (K1018, Apexbio, USA) kit. Groups of EcESCs at logarithmic growth phase were taken and cells were seeded into 96-well culture plates at a density of 1 × 10^3^ cells/well, with 100 μL of medium containing 10% FBS added to each well. Cells were incubated for the indicated time, then 10 μL of CCK-8 solution was added to each well of the plate and incubated for an additional 4 h at 37 °C. After incubation, optical density (OD) values were measured at 450 nm, and absorbance values were recorded on day 1, day 2 and day 3. Cell growth curves were plotted with five parallel wells set up for each experiment.

### Transwell assay

For cell migration capacity analysis, 2 × 10^5^ transfected cells were suspended in serum-free DMEM (200 μL) and added to the apical chamber of a Transwell uncoated with Matrigel (356,234, BD Bioscience, San Jose, CA, USA).

For cell invasion analysis, Matrigel (356,234, BD Bioscience) was diluted (1:10) in serum-free DMEM and diluted Matrigel (100 μL) was added to the apical chamber of the Transwell and incubated for more than 30 min. Then 2 × 10^5^ transfected cells were then seeded into the apical chamber coated with Matrigel.

The basolateral chambers were both spiked with DMEM containing 10% FBS (600 μL) and then incubated at 37 °C in an incubator for 24 h. Cells were then fixed with 4% paraformaldehyde for 15 min and stained with crystal violet (0.1%) for 15 min. The stained positive cells were then observed using an inverted light microscope (CarlZeiss, Germany) and photographed for imaging, with ImageJ software used for positive cell counting.

### Dual luciferase reporter gene assay

The target binding sequence of AFAP1-AS1 to miR-15a-5p 3’untranslated region (UTR) was inserted into the psh-Check2 plasmid, wild type (WT-AFAP1-AS1), followed by the construction of a AFAP1-AS1 sequence mutation vector using a point mutation kit (Takara), as MUT-AFAP1-AS1. The target binding site of BCL9 to miR-15a-5p was inserted into the pGL3 promoter vector (WT-BCL9), followed by the construction of a BCL9 sequence mutagenesis vector (MUT-BCL9) using a point mutation kit (Takara).

The constructed vector was then co-transfected with miR-15a-5p mimic or miR-NC (50 nM; GenePharma) in ESC using Lipofectamine 2000 reagent (11,668,019, Thermo Fisher Scientific), respectively. At 48 h post-transfection, relative luciferase activity was measured using the Dual Luciferase Reporter Assay Kit (E1910; Promega, Madison, WI, USA), and firefly luciferase activity was measured in the Dual Luciferase Reporter Assay System (Promega) with renilla luciferase activity used as an internal reference.

### RNA-pull down assay

Cells were transfected with biotin-labeled NC-Bio, AFAP1-AS1(MUT)-Bio, and AFAP1-AS1(WT)-Bio (50 nM each). After 48 h of transfection, cells were collected and washed with PBS. Cells were then incubated with specific cell lysis buffer (Ambion, Austin, TX, USA) for 10 min, after which 50 mL of sample cell lysis buffer was dispensed. The residual lysate was incubated with M-280 streptavidin magnetic beads (Sigma-Aldrich) pre-coated with RNase-free and yeast tRNA (Sigma-Aldrich) for 3 h at 4 °C. RNA was extracted and detected by RT-qPCR.

### RNA immunoprecipitation (RIP)

Cells were lysed in an ice bath for 5 min with lysis buffer and enzyme inhibitor (1,111,111, Roche, Germany), centrifuged at 14,000 rpm for 10 min at 4 °C to remove the supernatant. The binding of AFAP1-AS1 to miR-15a-5p protein was then detected using a RIP kit (Millipore, Billerica, MA, USA). One third of the cell extracts was removed as input and the remaining two thirds were incubated with antibodies for co-precipitation, respectively. Specifically, cells were mixed with 5 μg of antibody AGO2 (ab32381, 1/50, Abcam) at room temperature for 30 min, while IgG (ab109489, 1/100, Abcam) and magnetic beads were resuspended in 100 μL of RIP Wash Buffer. The magnetic bead-antibody complex was washed and resuspended in 900 μL of RIP Wash Buffer and incubated overnight at 4 °C with 100 μL of cell extract. Samples of the co-precipitation reaction system were placed on magnetic base to collect the bead-protein complexes. The co-precipitation reaction system samples and the Input were digested with proteinase K and RNA was extracted.

### Immunohistochemistry (IHC)

Paraffin sections of nude mouse endometriotic tissue were placed in a thermostat and stored at 60 °C for 2 h. Sections were dewaxed with xylene and hydrated with graded ethanol (100, 95, 85, and 70%). Then the sections were soaked in citrate buffer (0.01 mol/L, pH = 6.0) and heated at 95–100 °C for 30 min for antigen repair. Sections were washed with PBS and incubated with 0.5% TritonX100 for 30 min, followed by incubation with BCL9 primary antibody (1/100, 22,947–1-AP, Proteintech) overnight at 4 °C. Subsequently, sections were incubated with HRP-labelled goat anti-rabbit IgG secondary antibody (1:500; Life Technologies, Carlsbad, CA, USA) for 1 h at room temperature, followed by treatment with diaminobenzidine solution for 3–5 min. Sections were counterstained with hematoxylin for 1–3 min, dehydrated, and mounted with neutral balsam. A brown chromogen on the membrane indicates a positive immunoreaction. Images were visualized using a Nikon ECLIPSE Ti microscope (Fukasawa, Japan) system, and five high magnification fields were randomly selected during analysis to calculate the rate of positive cells out of 100 cells per field.

### Establishment of EMS nude mice model

Twenty Balb/c female nude mice (6–8 weeks, Kaixue Biotech, Shanghai, China) were housed in a sterile environment for 2 weeks in adaptive housing, with a light/dark cycle (12 h/12 h) at 23–25 °C. Ectopic endometrial tissues were obtained from 10 EMS patients, and endometrial fragments were resuspended in PBS and injected intraperitoneally into anesthetized nude mice through a 19-gauge needle (all patients’ tissue fragments were mixed well and each nude mouse was injected with tissues containing approximately 30 mg/0.2 mL of PBS). After 7 days of endometrial tissue implantation (day 7 of moulding), the nude mice were randomly treated with 10 mice in each treatment.

After EcESCs were transfected with plasmids of sh-NC and sh-AFAP1-AS1, the Exo was extracted from the culture medium supernatant separately and resuspended in PBS to a concentration of 30 μg/200 μL. Ten nude mice were injected intraperitoneally with sh-AFAP1-AS1-enriched Exo (Exo-sh-AFAP1-AS1) every 2 days; the other ten nude mice were injected intraperitoneally with sh-NC-enriched Exo (Exo-sh-NC) as control. After 24 h of the last injection (day 14 of modeling), the nude mice were euthanized and endometriotic tissues were collected for subsequent experimental analysis. The animal experiment protocol was approved by the Animal Ethics Committee of Second Xiangya Hospital, Central South University.

### Statistical analysis

All data were processed using SPSS 21.0 statistical software (SPSS, Inc., Armonk, NY, USA) and GraphPad Prism7. Measurement data derived from three times of experiments were expressed as mean ± standard deviation. Comparisons between two groups were made using independent samples *t*-test, and comparisons among multiple groups were performed by one-way analysis of variance (ANOVA). Cell proliferation at different times was performed by two-way ANOVA. A *p* < 0.05 indicates that the difference is statistically significant.

## Results

### AFAP1-AS1 is highly expressed in EMS tissues and cells, and Exo from EcESCs delivers AFAP1-AS1 to EcESCs

**Fig. 1:**
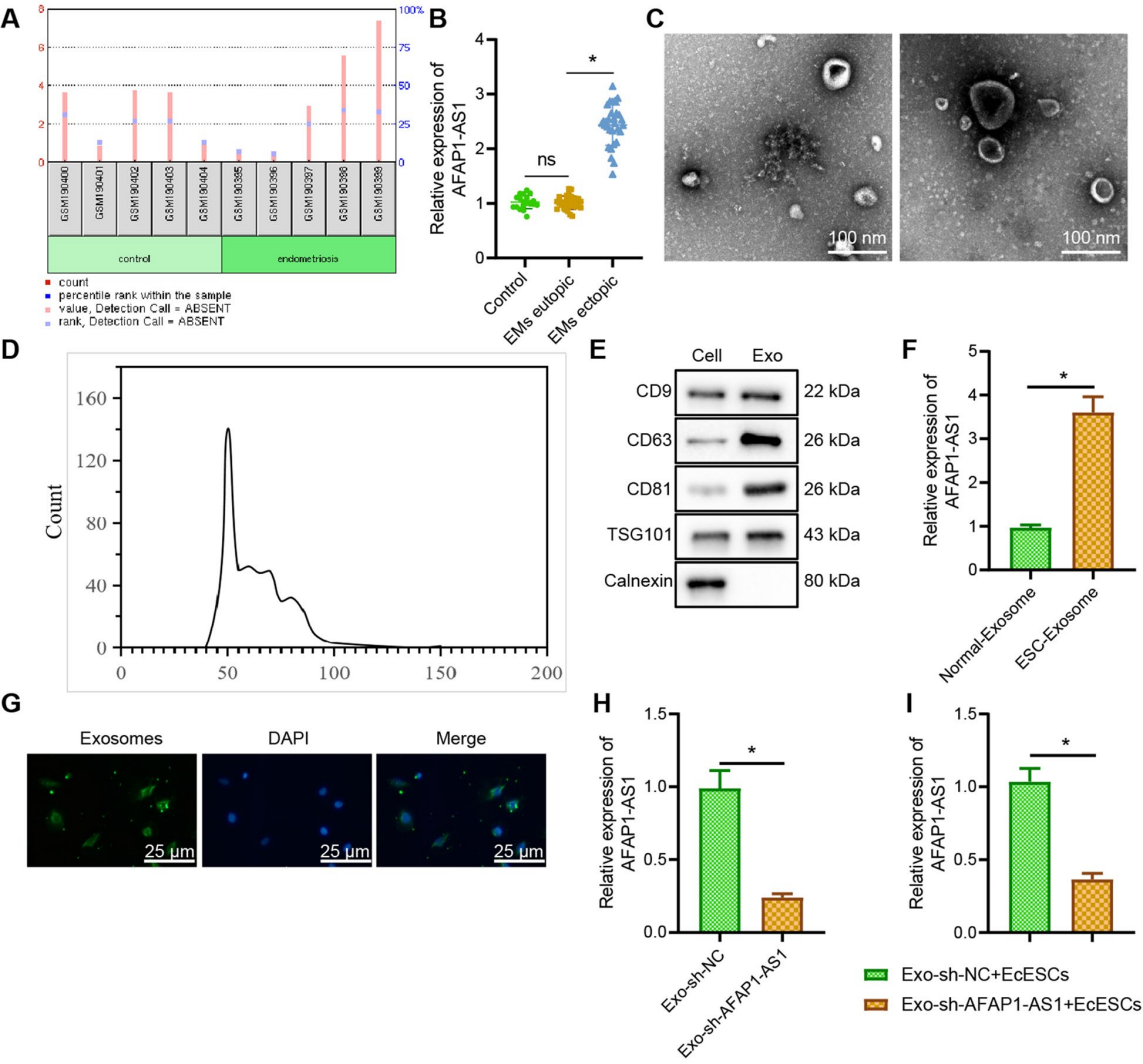
Identification of Exo and expression of AFAP1-AS1 in EcESCs and Exo. **A** Expression of AFAP1-AS1 in EMS patients and non-EMS patients in microarray GSE7846. **B** RT-qPCR to detect the expression of AFAP1-AS1 in normal endometrial tissues (*n* = 20), EMS eutopic endometrial tissues (*n* = 30), and ectopic endometrial tissues (*n* = 30). **C** TEM to observe morphology of Exo. **D** NTA to determine the particle size of normal and ESC-Exo. **E** Western blot analysis to detect protein expression of CD9, CD63, CD81, Tsg101, and Calnexin. **F** RT-qPCR to study AFAP1-AS1 in Normal and EcESCs-Exo. **G** Fluorescence microscopy to assess of PKH67-labelled Exo internalization in EcESCs. **H** RT-qPCR to detect AFAP1-AS1 expression in Exo after silencing AFAP1-AS1 in EcESCs. **I** RT-qPCR to monitor the AFAP1-AS1 expression in EcESCs after co-culture of Exo with EcESCs. * *p* < 0.05 indicates the comparison between two groups; ns *p* > 0.05 indicates the comparison between two groups

Differential analysis of microarray GSE7846 revealed significantly higher AFAP1-AS1 expression in EMS than in non-EMS controls (Fig. 1A). Among 30 collected EMS eutopic and ectopic endometrial tissues, as well as 20 normal endometrial tissues (control), the expression of AFAP1-AS1 in the EMS ectopic tissues was distinctly higher (Fig. 1B). Therefore, we speculated that Exo secreted by EcESCs could influence EMS disease progression through the delivery of AFAP1-AS1.

The ESCs were successfully isolated, which were in spindle-shape and expressed Vimentin protein (Supplementary Fig. 1A-B). Exo in the culture supernatant of control and EcESCs was isolated and observed by TEM, which showed a typical Exo structure including double-membrane vesicles around 150 nm (Fig. 1C). NTA analysis further confirmed that the size of Exo was mainly around 100–50 μm (Fig. 1D). CD9, CD63, CD81, and Tsg101 proteins were not expressed or little expressed in the cells but highly expressed in Exo. The endoplasmic reticulum membrane protein Calnexin was highly expressed in the cells but not or little expressed in Exo (Fig. 1E). AFAP1-AS1 was highly expressed in Exo from EcESCs (Fig. 1F). The above results indicated that we successfully extracted Exo and AFAP1-AS1 was highly expressed in Exo.

To investigate whether AFAP1-AS1 was delivered via Exo, we labelled EcESCsExo with PKH67 staining and then co-incubated Exo with EcESCs for 48 h. A green signal was seen in EcESCs by fluorescence microscopy (Fig. 1G), showing that Exo could be internalized by EcESCs. EcESCs transfected with sh-AFAP1-AS1 decreased the expression of AFAP1-AS1 in Exo (Fig. 1H). Meanwhile, the Transwell apical chamber was added with EcESCs treated with sh-AFAP1-AS1, while the basolateral chamber was added with EcESCs. RT-qPCR showed that the EcESCs with Exo-sh-AFAP1-AS1 showed reduced AFAP1-AS1 expression (Fig. 1I). The above results suggest that Exo can carry AFAP1-AS1 to EcESCs and silenced AFAP1-AS1 can downregulate AFAP1-AS1 expression in Exo as well as in EcESCs.

### Exo-sh-AFAP1-AS1 inhibits the proliferation, migration, and invasion of EcESCs

**Fig. 2:**
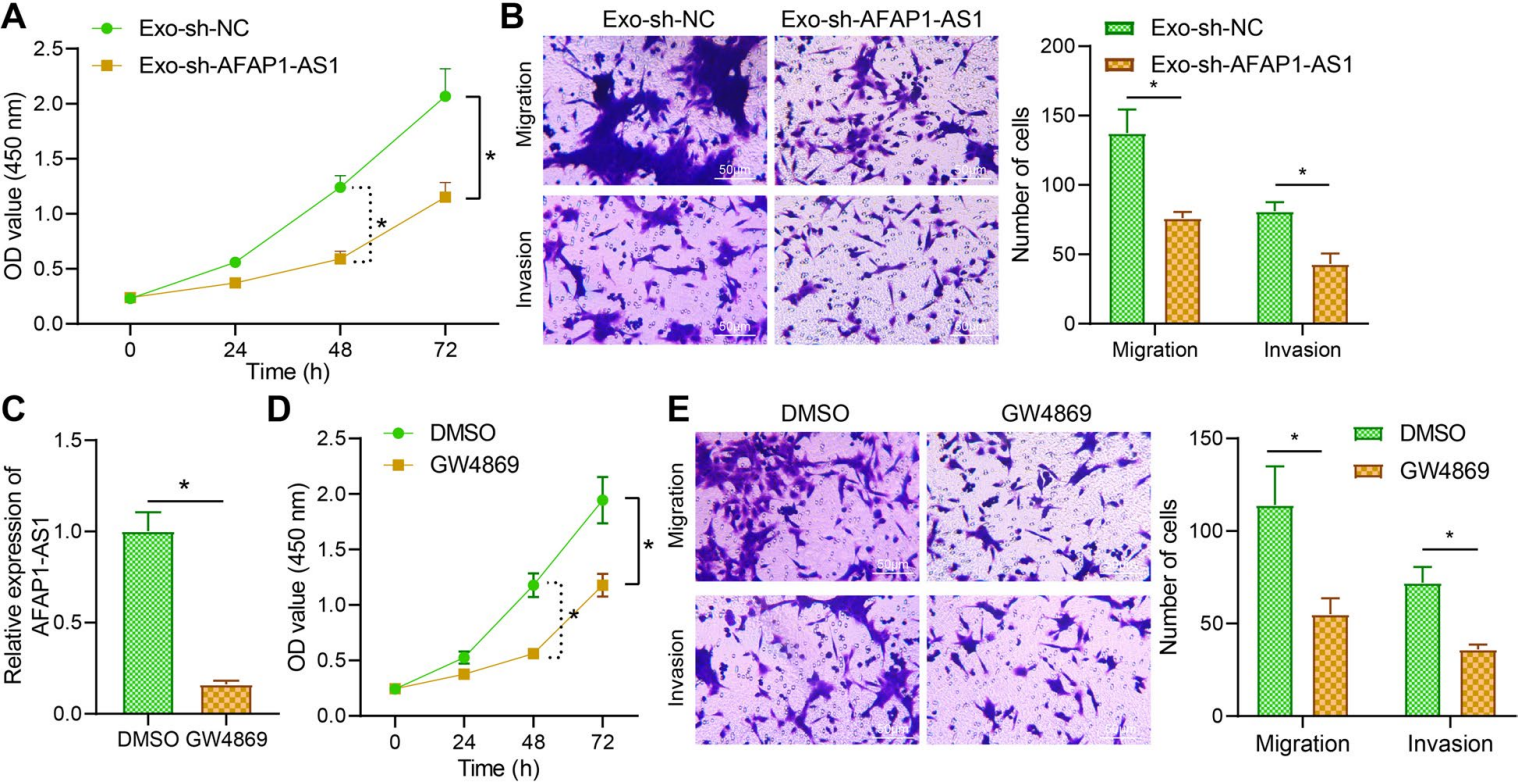
Effect of Exo-sh-AFAP1-AS1 on the proliferation, migration and invasion ability of EcESC. **A** CCK-8 to detect the effect of silencing AFAP1-AS1 in Exo on the proliferation of EcESCs. **B** Transwell to analyze the effect of silencing AFAP1-AS1 in Exo on the migration and invasion of EcESCs. **C** RT-qPCR to assess the effect of GW4869 on the expression of AFAP1-AS1 in EcESCs. **D** CCK-8 to detect the effect of GW4869 on the proliferation of EcESCs. **E** Transwell to detect the effect of GW4869 on the migration and invasion of EcESCs. * *p* < 0.05 indicates the comparison between two groups

The effect of AFAP1-AS1 in Exo on the proliferation, migration and invasion ability of EcESCs was further studied. In Exo with sh-AFAP1-AS1, a slower proliferation rate and a lower number of migrating and invading cells were detected (Fig. 2A, B).

Moreover, the GW4869-treated EcESCs reduced AFAP-AS1 expression (Fig. 2C), accompanied with inhibited proliferation of EcESCs and reduced number of migrated and invasive cells compared to the DMSO treatment (Fig. 2D, E). The above results indicated that Exo-sh-AFAP1-AS1 could inhibit the proliferation, migration and invasion of EcESCs.

### Silencing of AFAP1-AS1 upregulates miR-15a-5p to inhibit the proliferation, migration, and invasion of EcESCs

**Fig. 3:**
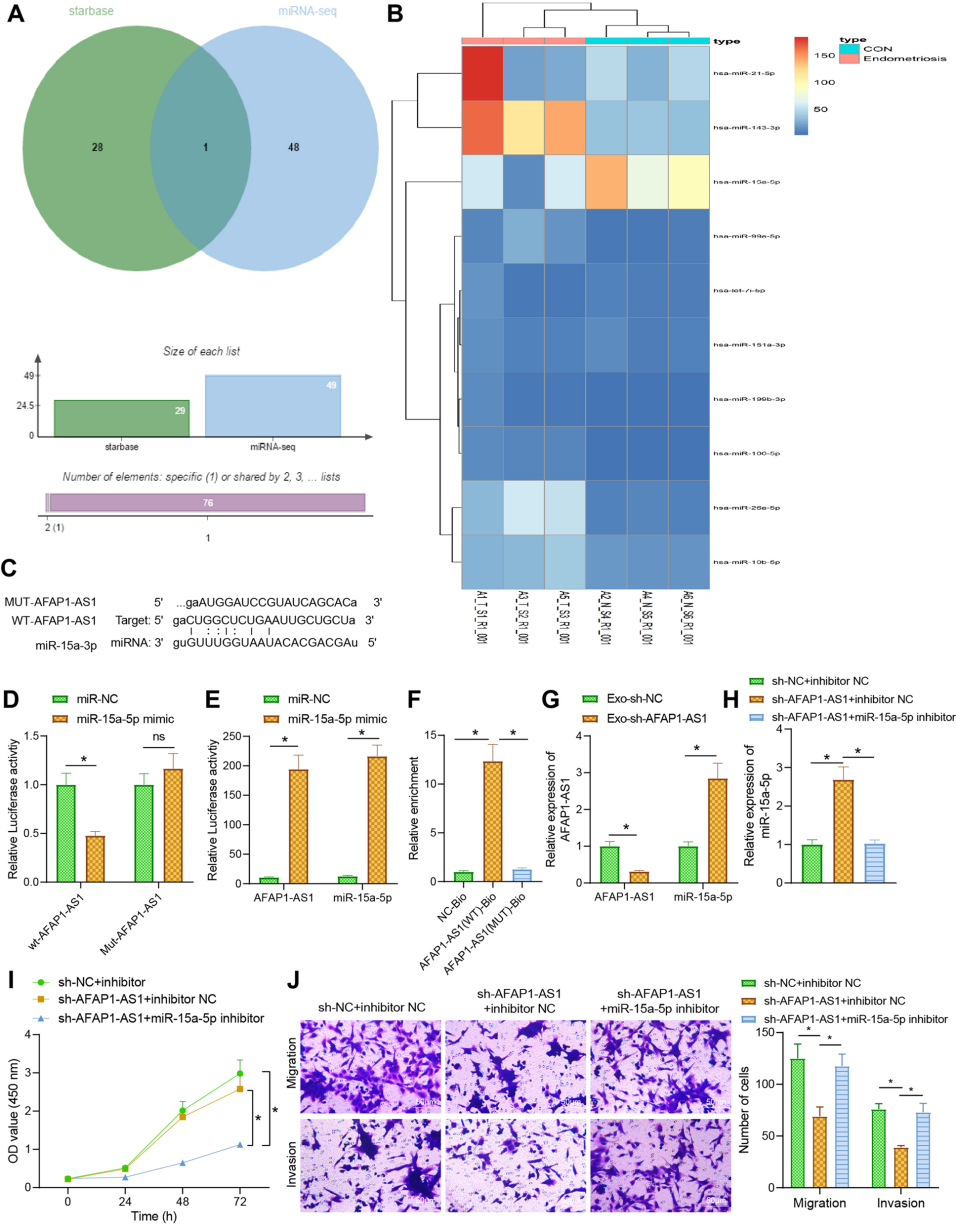
Effect of AFAP1-AS1 binding to miR-15a-5p on the proliferation, migration and invasion ability of EcESCs. **A** Venn diagram of the intersection of Starbase database predicted binding miRNAs for AFAP1-AS1 and miRNA second generation sequencing analysis of differential miRNAs. **B** Heat map of the top 10 differential miRNAs for miRNA second generation sequencing analysis. **C** Predicted binding sites for AFAP1-AS1 to miR-15a-5p via LncBase. **D** Dual luciferase reporter gene assay to verify the targeted binding of AFAP1-AS1 to miR-15a-5p. **E** RIP assay to detect the enrichment of AFAP1-AS1 and miR-15a-5p in IgG and Ago2. **F** RNA pull down assay to detect the binding of AFAP1-AS1 to miR-15a-5p. **G** RT-qPCR to detect the changes of AFAP1-AS1 and miR-15a-5p expression after silencing of AFAP1-AS1. **H** RT-qPCR to detect the changes of miR-15a-5p expression in differently treated EcESCs. **I** CCK-8 to detect the changes of cell proliferation ability. **J** Transwell assay to detect the changes of cell migration and invasion ability. * *p* < 0.05 indicates the comparison between two groups; ns *p* > 0.05 indicates the comparison between two groups

To explore the molecular mechanism downstream of AFAP1-AS1, the miRNAs downstream of AFAP1-AS1 were predicted by Starbase database (http://starbase.sysu.edu.cn/) and miRNA second-generation sequencing. miR-15a-5p was the intersected result (Fig. 3A). The results of second-generation sequencing indicated that miR-15a-5p was poorly expressed in endometriotic samples (Fig. 3B).

The binding site of AFAP1-AS1 to miR-15a-5p was predicted through the LncBase database (Fig. 3C), and subsequently, miR-15a-5p was overexpressed by plasmid of miR-15a- 5p mimic in EcESCs. The luciferase activity was significantly reduced in the wt-AFAP1-AS1 co-transfected with miR-15a-5p mimic (Fig. 3D), showing that miR-15a-5p can bind to AFAP1-AS1. The expression of AFAP1-AS1 and miR-15a-5p was increased in the Ago2, indicating that Ago2 was able to enrich AFAP1-AS1 and miR-15a-5p (Fig. 3E). Meanwhile, miR-15a-5p was enriched in AFAP1-AS1(WT)-Bio samples (Fig. 3F). Subsequently, we silenced AFAP1-AS1 in EcESCs, and AFAP1-AS1 expression was reduced, while miR-15a-5p expression was increased after silencing of AFAP1-AS1 (Fig. 3G). These results suggest that AFAP1-AS1 can bind to miR-15a-5p and downregulate its expression.

We further silenced miR-15a-5p by plasmid of miR-15a-5p inhibitor based on silencing of AFAP1-AS1. miR-15a-5p expression was elevated after AFAP1-AS1 knockdown, which was then reduced after miR-15a-5p was silenced (Fig. 3H). Silencing of AFAP1-AS1 also inhibited cell proliferation, migration, and invasion, and further silencing of miR-15a-5p promoted cell proliferation, migration, and invasion (Fig. 3I, J). The above results suggest that silenced AFAP1-AS1 can upregulate miR-15a-5p, thereby inhibiting the proliferation, migration, and invasion of EcESCs.

### miR-15a-5p inhibits and targets BCL9 expression in EcESCs

**Fig. 4:**
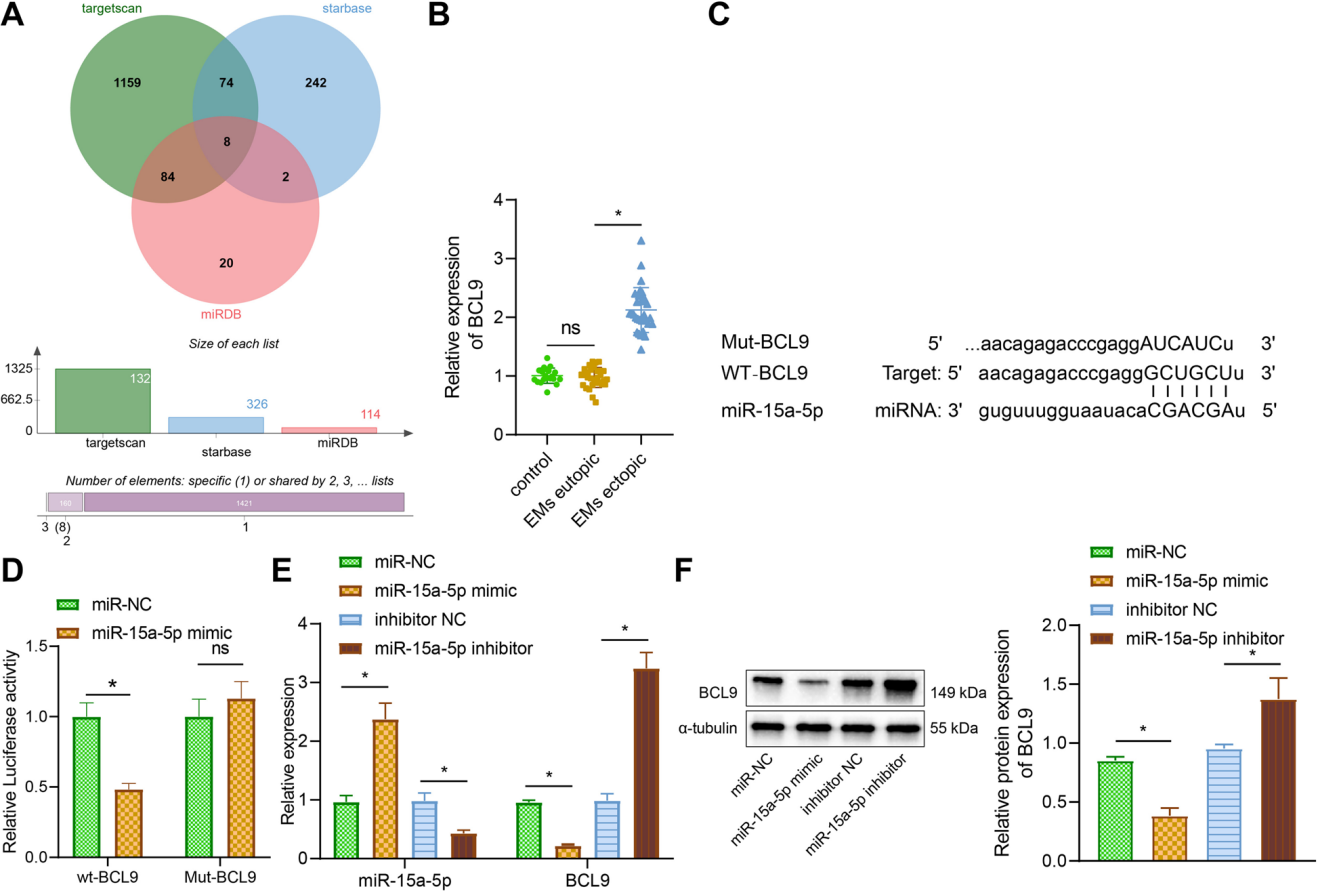
Regulation of BCL9 by miR-15a-5p in EcESCs. **A** Venn diagram of the target genes of miR-15a-5p predicted by Starbase, TargetScan and miRDB databases. **B** RT-qPCR to detect BCL9 expression in 30 EMS eutopic and ectopic tissues, and 20 normal endometrial tissues. **C** TargetScan online prediction of miR-15a-5p. **D** Dual luciferase reporter gene assay to verify the target binding of miR-15a-5p to BCL9. **E** RT-qPCR to detect the expression of miR-15a-5p and BCL9 after overexpression or silencing of miR-15a-5p. **F** Western blot analysis to detect the protein expression of BCL9 after overexpression or silencing of miR-15a-5p, **F** Western blot analysis to detect the protein expression of BCL9 after overexpression or silencing of miR-15a-5p. * *p* < 0.05 indicates the comparison between two groups; ** *p* < 0.01 indicates the comparison between two groups; ns *p* > 0.05 indicates the comparison between two groups

The Starbase database, TargetScan database (http://www.targetscan.org/vert_72/) and miRDB database (http://mirdb.org/) were used to predict miR-15a -5p target genes, eventually identifying AKT3, AK4, AGO4, SYDE2, BCL9, CMPK1, CSDE1, and TGFBR3 as possible target genes for miR-15a-5p (Fig. 4A).

BCL9 expression was significantly higher in the EMS ectopic tissues (Fig. 4B). miR-15a-5p was found to have a binding site to BCL9 by online prediction in the TargetScan database (Fig. 4C). Therefore, we speculated that AFAP1-AS1 may affect BCL9 expression by binding to miR-15a-5p. The luciferase activity was significantly reduced in the WT-BCL9 in cells co-transfected with miR-15a-5p mimic, whereas no significant change in luciferase was observed in the MUT-BCL9 in cells co-transfected with miR-15a-5p mimic (Fig. 4D), suggesting that miR-15a-5p could target and bind to BCL9.

In addition, we overexpressed or inhibited miR-15a-5p in EcESCs. miR-15a-5p expression was upregulated and BCL9 expression was downregulated after overexpression of miR-15a-5p, while miR-15a-5p expression was downregulated and BCL9 expression was upregulated after silencing of miR-15a-5p (Fig. 4E, F). These results suggest that miR-15a-5p can target and inhibit BCL9 expression in EcESCs.

### miR-15a-5p targets downregulation of BCL9 expression to inhibit proliferation, migration and invasion of EcESCs

**Fig. 5:**
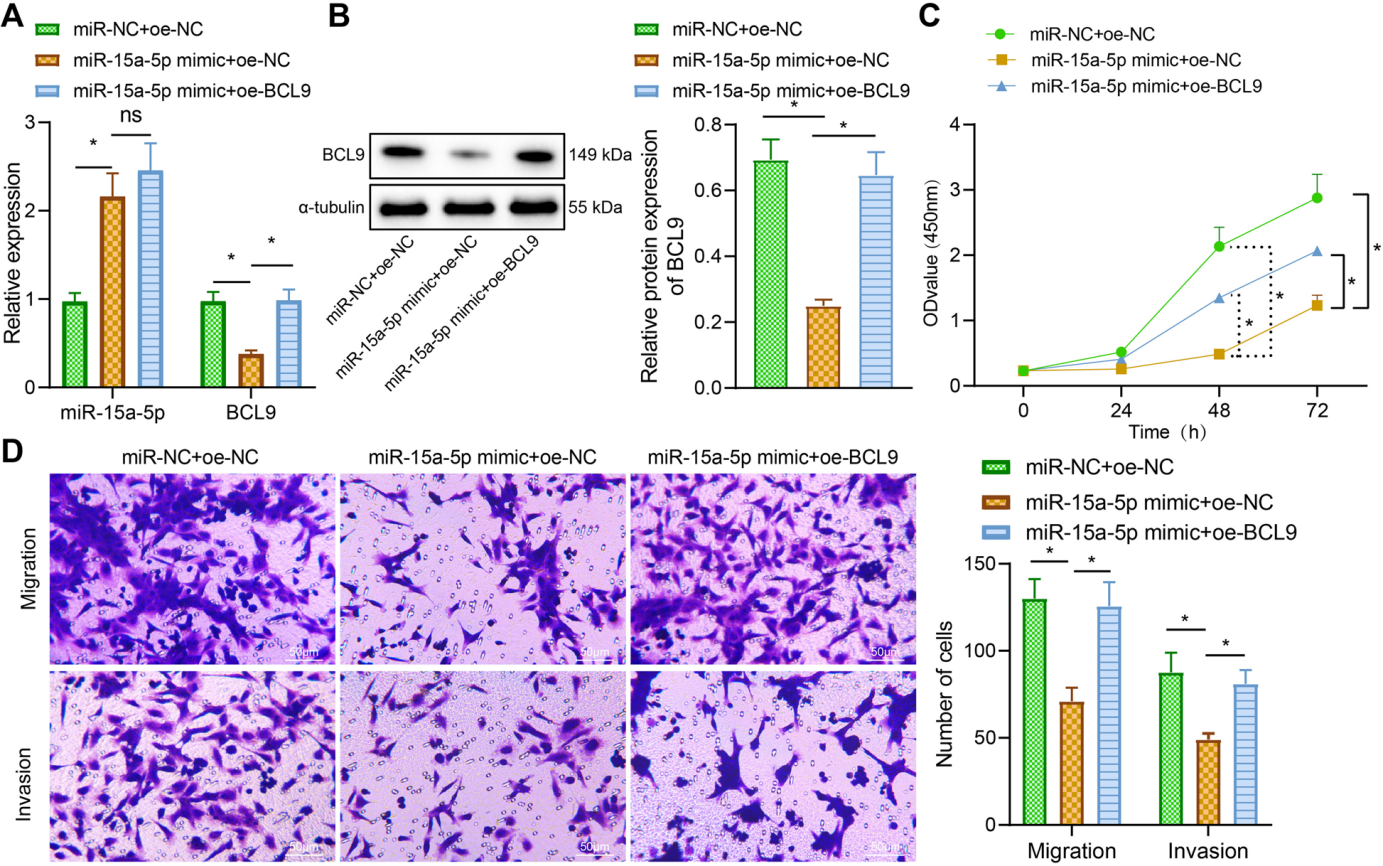
Effect of miR-15a-5p-targeted regulation of BCL9 on the proliferation, migration and invasion ability of EcESCs. **A** RT-qPCR to detect the effect of overexpression of miR-15a-5p and BCL9 on the expression of miR-15a-5p and BCL9. **B** Western blot analysis to detect the effect of overexpression of miR-15a-5p and BCL9 on the protein expression of BCL9. **C** CCK-8 to detect the proliferation of differently treated cells. **D** Transwell assay to detect the migration and invasion ability of cells (scale bar = 50 μm). * *p* < 0.05 indicates the comparison between two groups; ** *p* < 0.01 indicates the comparison between two groups; ns *p* > 0.05 indicates the comparison between two groups

Next, we investigated whether miR-15a-5p affected the proliferation, migration and invasion of EcESCs by regulating BCL9. miR-15a-5p expression increased and BCL9 expression decreased after upregulation of miR-15a-5p. After overexpression of both miR-15a-5p and BCL9, miR-15a-5p expression did not significantly changed while BCL9 expression increased (Fig. 5A, B).

Overexpression of miR-15a-5p by miR-15a-5p mimic inhibited cell proliferation, migration, and invasion, while further overexpression of BCL9 promoted cell proliferation, migration, and invasion (Fig. 5C, D). These results suggest that miR-15a-5p targets down-regulation of BCL9 expression, which in turn inhibits the proliferation, migration and invasion of EcESCs.

### AFAP1-AS1 promotes the proliferation, migration and invasion of EcESCs through miR-15a-5p/BCL9

**Fig. 6:**
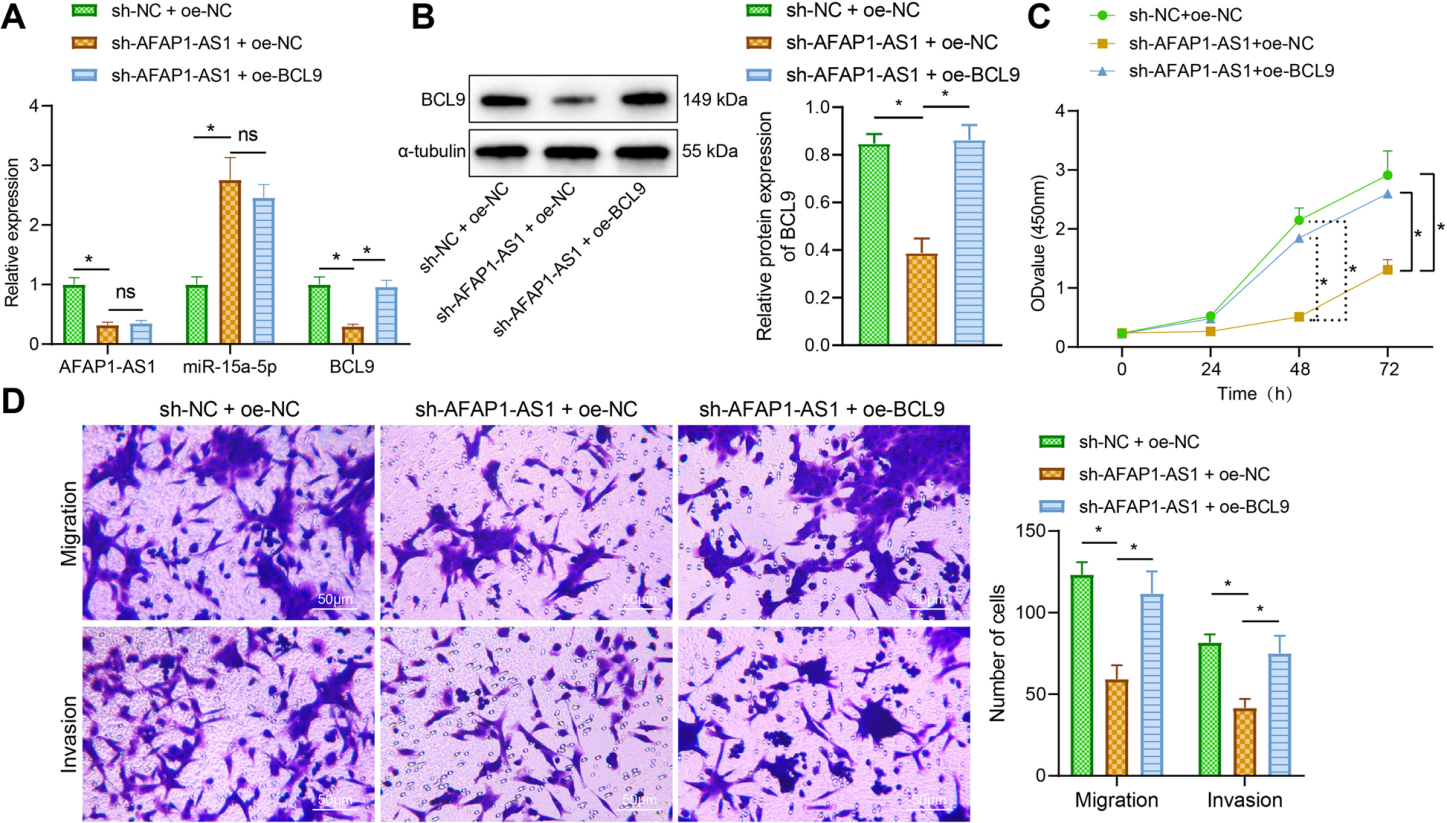
Effect of AFAP1-AS1/miR-15a-5p/BCL9 on the proliferation, migration and invasion ability of EcESCs. **A** RT-qPCR to detect the effect of inhibition of AFAP1-AS1 and overexpression of BCL9 on the expression of AFAP1-AS1, miR-15a-5p and BCL9. **B** Western blot analysis to detect the effect of inhibition of AFAP1-AS1 and overexpression of BCL9 on the expression of BCL9. **C** CCK-8 to detect the changes in cell proliferation. **D** Transwell assay to detect changes in cell migration and invasion ability (scale bar = 100 μm). * *p* < 0.05 indicates the comparison between two groups; ns *p* > 0.05 indicates the comparison between two groups

Whether AFAP1-AS1 mediated miR-15a-5p/BCL9 and thus affected the proliferation, migration and invasion of EcESCs were subsequently researched. After AFAP1-AS1 knockdown, AFAP1-AS1 and BCL9 expression was downregulated, while miR-15a-5p expression was upregulated. Further overexpression of BCL9 did not affect AFAP1-AS1 and miR-15a-5p expression, while BCL9 expression was upregulated (Fig. 6A, B), suggesting that AFAP1-AS1 could upregulate BCL9 by binding to miR-15a-5p.

Meanwhile, inhibition of AFAP1-AS1 suppressed cell proliferation, migration, and invasion, while further overexpression of BCL9 promoted cell proliferation, migration, and invasion (Fig. 6C, D). The above results suggest that AFAP1-AS1 binds to miR-15a-5p to upregulate BCL9, which in turn promotes proliferation, migration, and invasion of ESCs.

### Exo-sh-AFAP1-AS1 inhibits migration and invasion of ectopic endometrium in EMS mice via miR-15a-5p/BCL9

**Fig. 7:**
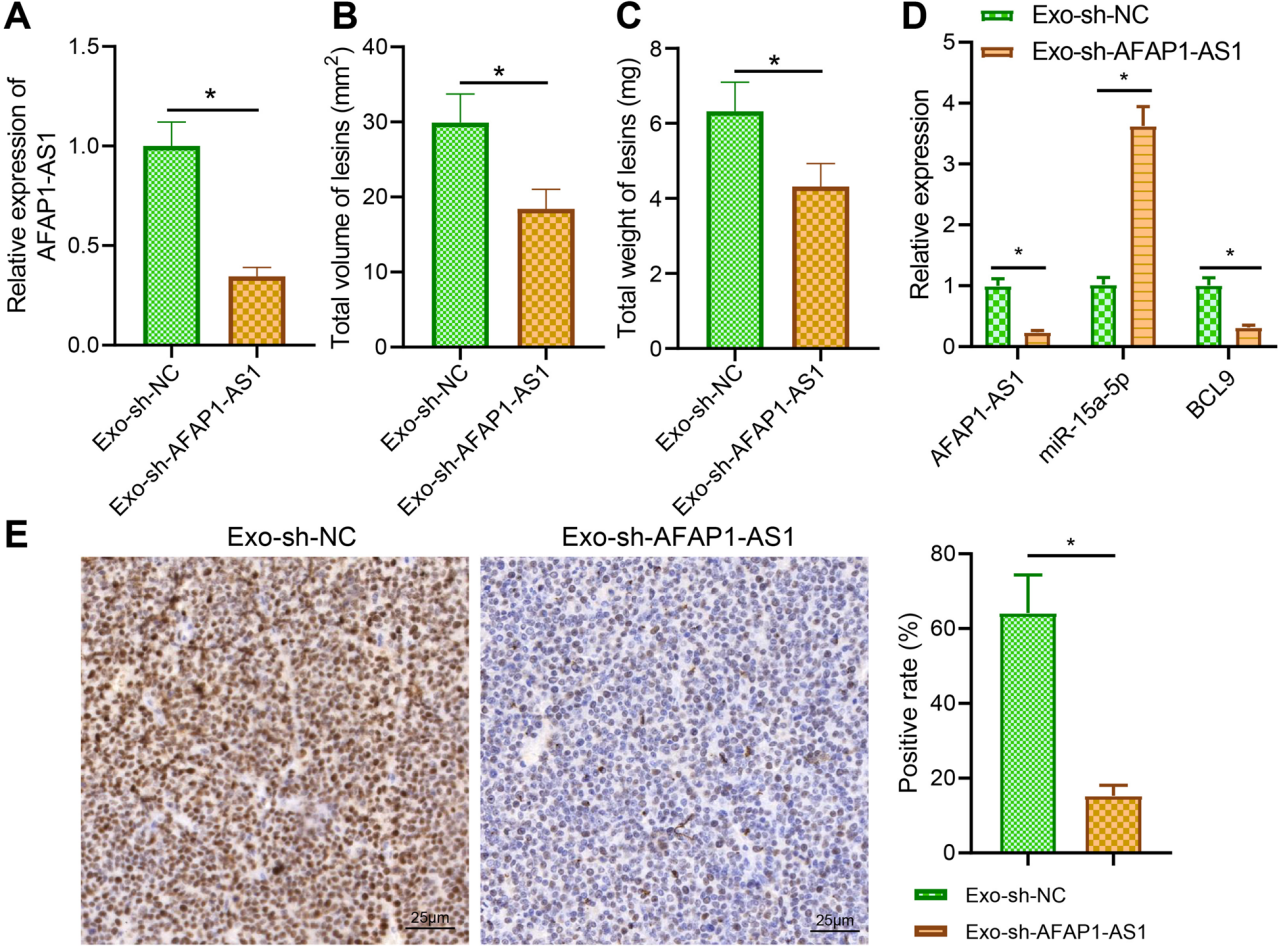
Effects of Exo-AFAP1-AS1/miR-15a-5p/BCL9 on EMS progression in nude mice. **A** RT-qPCR to detect the expression of AFAP1-AS1 in ESC-Exo after silencing of AFAP1-AS1. **B** Change in volume of ectopic lesions in EMS nude mice after Exo-sh-NC and Exo-sh-AFAP1-AS1 treatment. **C** Change in weight of ectopic lesions in EMS after Exo-sh-NC and Exo-sh-AFAP1-AS1 treatment. **D** RT-qPCR to analyze the expression of AFAP1-AS1, BCL9 and miR-15a-5p in the ectopic tissues of EMS mice after Exo-sh-NC and Exo-sh-AFAP1-AS1 treatment. **E** IHC to detect the expression of AFAP1-AS1, BCL9, and miR-15a-5p in the ectopic tissues of EMS mice after Exo-sh-NC and Exo-sh-AFAP1-AS1 treatment. * *p* < 0.05 indicates the comparison between two groups. A total of 10 nude mice per treatment

Finally, we explored the effect of Exo-derived AFAP1-AS1/miR-15a-5p/BCL9 on ectopic tissue formation in EMS mice by in vivo experiments. Exo was extracted from EcESCs transfected with sh-NC and sh-AFAP1-AS1, and AFAP1-AS1 content in Exo isolated from the sh-AFAP1-AS1 group (Exo-sh-AFAP1-AS1) was significantly lower (Fig. 7A).

After removal of the endometrial lesions from the EMS mice, the ectopic lesions in the Exo-sh-AFAP1-AS1-treated mice were significantly smaller in size and lower in weight (Fig. 7B, C). miR-15a-5p was significantly upregulated and BCL9 was downregulated in the Exo-sh-AFAP1-AS1 treated mice (Fig. 7D). BCL9 expression was reduced in the Exo-sh-AFAP1-AS1 treated mice (Fig. 7E). The above results indicate that Exo-sh-AFAP1-AS1 could inhibit the migration and invasion of ectopic endometrium in EMS mice through miR-15a-5p/BCL9.

## Discussion

Exosomal lncRNA and its related networks are associated with EMS, which indicates a novel molecular mechanism of EMS and offers new strategies for EMS treatment [15]. This study elucidates a potential crosstalk between EcESCs via Exo in EMS, suggests a novel mechanism for ESC migration/invasion from the perspective of the “Exo transfer of lncRNAs” and highlights the potential of AFAP1-AS1 as a biomarker for EMS.

At the beginning of our study, we performed bioinformatics study, which showed that AFAP1-AS1 was highly expressed in EMS. Consistent with our study, it has been documented that the expression of AFAP1-AS1 in EMS was significantly higher than that in normal control [16]. AFAP-AS1 could affect the proliferation, migration, and invasion of ESCs, and contributed to ESC angiogenesis as well as tumor metastasis, which accelerates the angiogenesis and invasion of endometrial carcinoma [9]. We further detected that in successfully-isolated Exo from EcESCs, AFAP1-AS1 was also highly expressed. The successful isolation of Exo was evidenced by morphology observation and positive expressions of specific biomarkers, TSG101, CD9, CD63, and CD81 [17]. Our results showed that Exo could transfer AFAP1-AS1 to EcESCs. Likewise, Exo could be uptaken by the ambient ESCs, and the exosomal lncRNA deleted in lymphocytic leukemia1 (DLEU1) exerts promoting effect on tumor cells in EMS [18]. These references supported our experimental results that AFAP1-AS1 played oncogenic role in EMS, which could be carried to EcESCs by Exo derived from ESCs.

We further demonstrated that AFAP1-AS1 could negatively regulate miR-15a-5p expression in EMS. miR-15a-5p was poorly expressed in EMS. Similarly, level of exosomal miR-214-3p in EMS patients’ serum was lower than that of non-EMS patient [19]. Moreover, overexpression of miR-214 offers an alternative therapeutic approach for EMS fibrosis [20]. Consistent with our finding, the endometrial tissue showed significantly lower expression of miR-15a-5p [21]. Although lncRNA could interact with miR-15a-5p in diseases, such as the regulatory mechanism of lncRNA CERS6 antisense RNA 1 and miR-15a-5p in pancreatic cancer [22], the relationship between AFAP1-AS1 and miR-15a-5p in EMS is rarely reported in existing works. miR-30s can affect the activity of Wnt/β-catenin signaling pathway by negatively regulating BCL9, which in turn inhibits malignant behaviors such as proliferation and migration of multiple myeloma cells [23], but few study has yet shown whether miR-15a-5p targets BCL9 and whether it has a regulatory effect on EMS. However, we proposed that miR-15a-5p could suppress the expression of its target gene BCL9. The other member in BCL family, BCL6 protein expression was significantly higher in the secretory phase of EMS patients [24]. More importantly, BCL9 is one of the oncogenes associated with EMS [14]. The publishing studies cooperatively explained the mechanism of AFAP1-AS1/miR-15a-5p/BCL9 in EMS.

The function of AFAP1-AS1/miR-15a-5p/BCL9 in EcESCs and EMS mice was further explored. AFAP1-AS1 targeted upregulation of BCL9 by binding to miR-15a-5p, which promoted proliferation, migration, and invasion of EcESCs. The changes of migration and invasion of ESCs are largely correlated with the progression of EMS [25]. Specifically, suppressed proliferation, migration, and invasion capabilities of ESCs represent anti-tumor effect in EMS [26]. Additionally, in our EMS mice, Exo carrying AFAP1-AS1 affected EMS also through miR-15a-5p/BCL9, contributing changes of tumor weight and size in EMS mice.

## Conclusion

**Fig. 8:**
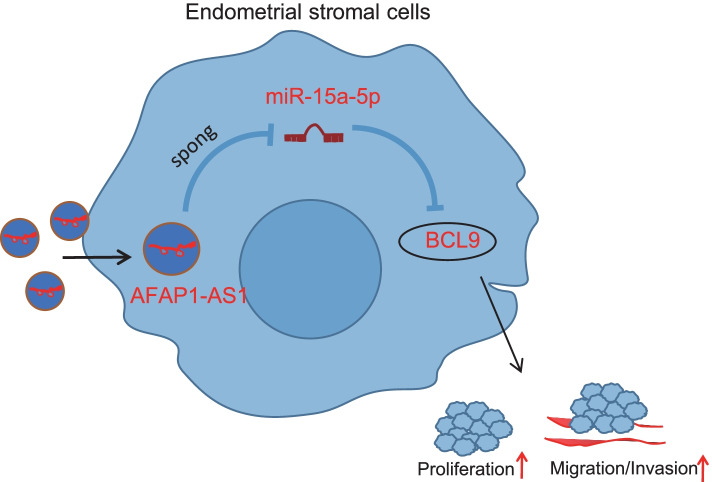
Schematic representation of the molecular mechanisms involved in the development of EMS by an ESCs-derived exosome-mediated AFAP1-AS1/miR-15a-5p/BCL9 axis

In conclusion, the high expression of AFAP1-AS1 in EMS was verified. AFAP1-AS1 expression was correlated with invasion, migration, and proliferation capabilities of EcESCs. This suggests that AFAP1-AS1 may serve as a new biomarker for the diagnosis of EMS prognosis. In addition, the silencing of AFAP1-AS1 might significantly suppress the proliferation, migration, and invasion of ESCs in cooperation with miR-15a-5p and BCL9 (Fig. 8). However, the specific mechanism awaits to be further investigated. A subsequent investigation of AFAP1-AS1 is needed and may improve the development of therapies for the prevention and treatment of EMS.

## Supplementary Information

**Additional file 1: Supplementary Figure 1.** Identification of various types of ESCs. A, Immunofluorescence to observe the morphology of each group of cells and the expression of Vimentin protein (scale bar = 200 μm). B, IHC to detect Vimentin protein expression in each group of cells (scale bar = 50 μm).

**Additional file 2: Supplementary Table 1.** Primer sequences for RT-qPCR.

## Data Availability

The datasets used and/or analyzed during the current study are availablefrom the corresponding author on reasonable request.

## Abbreviations

EMS: Endometriosis
Exo: Eexosome
AFAP1-AS1: Actin filament associated protein 1-Antisense RNA 1
ESCs: Endometrial stromal cells
NTA: Nanoparticle tracking analysis
IHC: Immunohistochemistry
PBS: Phosphate buffer saline
IgG: Munoglobulin G
TEM: Transmission electron microscopy
NTA: Nanoparticle tracking analysis
RIPA: Radio immunoprecipitation analysis
SDS-PAGE: Sodium dodecyl sulfate polyacrylamide gel electrophores
PVDF: Polyvinylidene fluoride
ECL: Enhanced chemiluminescence
CCK-8: Cell counting kit-8
