# Supplementary material for: Exosomal AFAP1-AS1 binds to microRNA-15a-5p to promote the proliferation, migration, and invasion of ectopic endometrial stromal cells in endometriosis
Source: Reprod Biol Endocrinol. 2022 May 5;20:77. doi: 10.1186/s12958-022-00942-1 (PMC9069797; doi:10.1186/s12958-022-00942-1)
Supplement: Supplementary file 2 — Additional file 2: Supplementary Table 1. Primer sequences for RT-qPCR. [file 12958_2022_942_MOESM2_ESM.docx]

**Supplementary Table 1.** Primer sequences for RT-qPCR

|  | Primer sequences |
| --- | --- |
| AFAP1-AS1 | F: 5’-AATGGTGGTAGGAGGGAGGA-3’ |
|  | R: 5’-CACACAGGGGAATGAAGAGG-3’ |
| GAPDH | F: 5’-GCACCGTCAAGGCTGAGAAC-3’ |
|  | R: 5’-ATGGTGGTGAAGACGCCAGT-3’ |
| miR-15a-5p (human) | F: 5’-GGCGGTAGCAGCACATCATG-3’ |
|  | R: 5’-GTGCAGGGTCCGAGGT-3’ |
| miR-15a-5p (mouse) | F: 5’-GTCCTCATCGCATACCATACA-3' |
|  | R: 5’-GCTGAAGTAAGGTTGGCAATA-3' |
| U6 (human) | F: 5’-CTCGCTTCGGCAGCACA-3' |
|  | R: 5’-AACGCTTCACGAATTTGCGT-3' |
| U6 (mouse) | F: 5’-GCTTGCTTCGGCAGCACATATAC-3' |
|  | R: 5’-TGCATGTCATCCTTGCTCAGGG-3' |

Notes: GAPDH, Glyceraldehyde-3-phosphate dehydrogenase; miR-15a-5p, microRNA-15a-5p, RT-qPCR, reverse transcription quantitative polymerase chain reaction; F, forward; R, reverse.
